# Secondhand smoke exposure in never-smokers: A cross-sectional and prospective cohort study of chronic kidney disease prevalence and all-cause mortality in affected patients

**DOI:** 10.1097/MD.0000000000050614

**Published:** 2026-09-18

**Authors:** Renjie Huang, Miao Huang, Rui Zhang

**Affiliations:** a The Second Clinical Medical College, Heilongjiang University of Chinese Medicine, Harbin, China; b Department of Andrology, The Second Affiliated Hospital of Heilongjiang University of Chinese Medicine, Harbin, China.

**Keywords:** all-cause mortality, chronic kidney disease, NHANES, secondhand smoke exposure

## Abstract

Chronic kidney disease (CKD) is a major global health burden, yet the association of secondhand smoke (SHS) exposure with CKD among never-smokers remains incompletely characterized. This cross-sectional and prospective cohort study analyzed 13,276 never-smoking adults from the National Health and Nutrition Examination Survey from 1999 to 2020. Multivariable logistic regression was used to evaluate CKD prevalence, and Cox proportional hazards models were used to evaluate all-cause mortality among participants with CKD. Sensitivity and subgroup analyses were conducted to assess the robustness of the findings. Moderate SHS exposure was associated with higher CKD prevalence (odds ratio = 1.37, 95% confidence interval [CI]: 1.07–1.76; *P* = .01). Among 1630 participants with CKD, moderate and heavy SHS exposure were associated with higher all-cause mortality (hazard ratio [HR] = 2.03 [95% CI: 1.23–3.36] and HR = 1.80 [95% CI: 1.11–2.93], respectively). Each doubling of serum cotinine concentration was associated with a 4% higher mortality hazard (HR = 1.04, 95% CI: 1.01–1.08; *P* = .02). Sensitivity analyses incorporating sampling weights and additional covariates supported the overall pattern of associations. Among never-smoking adults, moderate SHS exposure was associated with higher CKD prevalence; among those with CKD, higher SHS exposure was associated with greater all-cause mortality. These observational findings support further prospective investigation and reinforce the importance of minimizing SHS exposure.

## 1. Introduction

Chronic kidney disease (CKD) is a growing global public health problem.^[1]^ Progressive loss of kidney structure and function increases the risks of cardiovascular disease (CVD), end-stage kidney disease, and all-cause mortality.^[2,3]^ These outcomes impose substantial burdens on patients and health care systems.^[4]^ Identifying potentially modifiable exposures associated with CKD and its prognosis is therefore important for prevention and risk reduction.

Active smoking is an established adverse exposure across multiple organ systems, including the kidney.^[5,6]^ Health risks may also extend to environmental secondhand smoke (SHS) exposure. Evidence increasingly suggests an association between SHS exposure and CKD among never-smokers.^[7]^ Accurate exposure assessment is important because self-reported SHS exposure may be subject to misclassification. Cotinine, the major metabolite of nicotine, is a widely used biomarker of recent tobacco-smoke exposure.^[8,9]^ Its longer half-life than nicotine allows recent exposure to be characterized more reliably than by self-report alone.^[10]^ Serum cotinine has therefore been used extensively in epidemiologic studies of SHS exposure among non-active smokers.^[11]^

Previous studies have reported associations between SHS exposure and CKD,^[2]^ but uncertainty remains regarding exposure-level patterns and the prognostic relevance of SHS among people who already have CKD. We therefore used National Health and Nutrition Examination Survey (NHANES) data to examine the association of biomarker-defined SHS exposure with CKD prevalence among never-smoking adults and, among participants with CKD, with subsequent all-cause mortality.

## 2. Materials and methods

### 2.1. Study population

**Figure 1.**
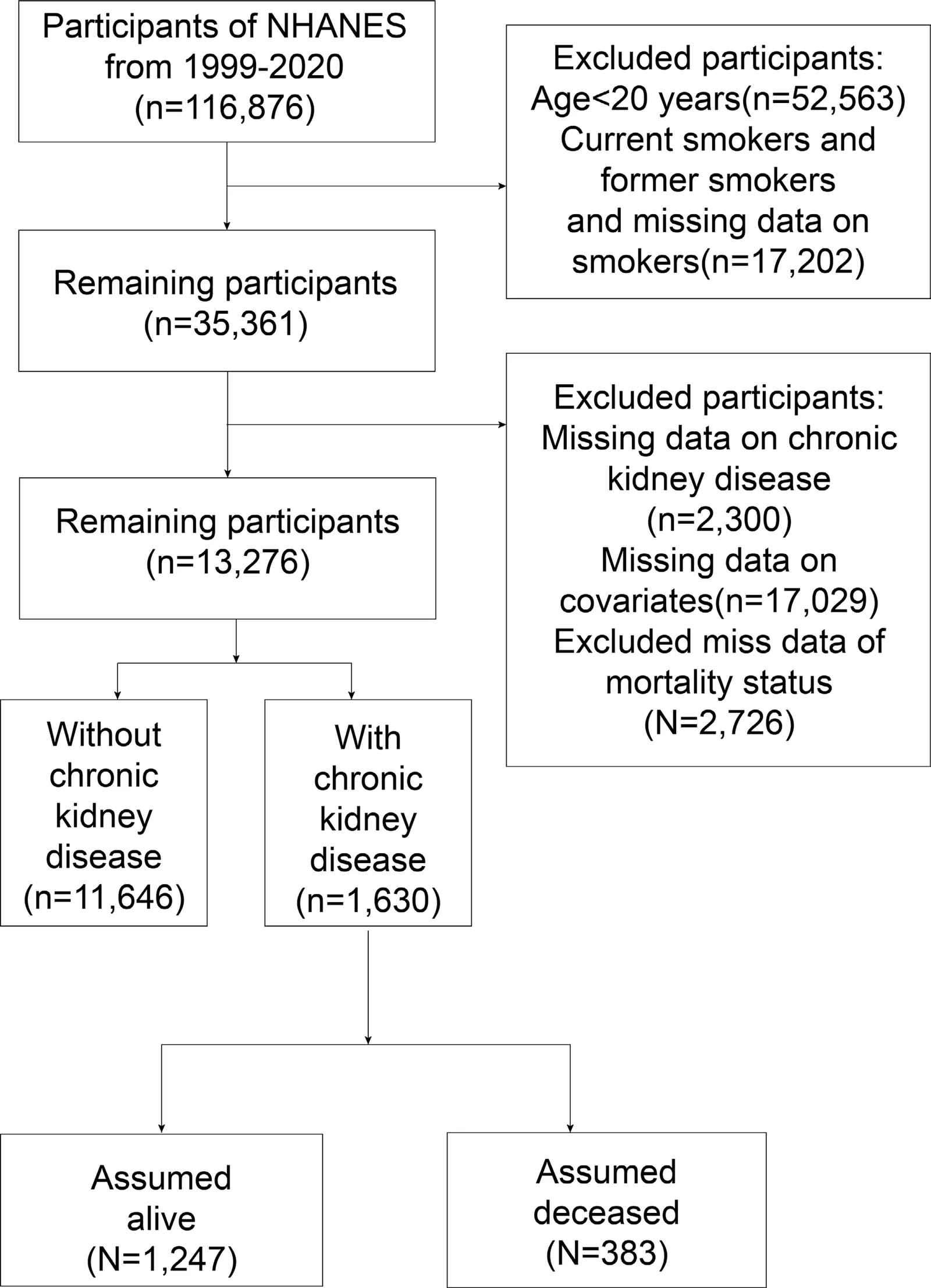
Flow diagram of participant screening and inclusion. NHANES = National Health and Nutrition Examination Survey.

This study used NHANES data from 1999 to 2020. NHANES is administered by the National Center for Health Statistics to assess the health and nutritional status of the noninstitutionalized US population. Public NHANES data and documentation are available at https://www.cdc.gov/nchs/nhanes/. The National Center for Health Statistics Research Ethics Review Board approved the NHANES protocols, and all participants provided written informed consent. This study was reported in accordance with the Strengthening the Reporting of Observational Studies in Epidemiology statement. Mortality data were obtained from the NHANES-linked National Death Index files through December 31, 2019. Follow-up was calculated from the NHANES examination date to the date of death or December 31, 2019, whichever occurred first. The median follow-up duration was 7.8 years. Participants aged 20 years or older with complete data on CKD, mortality, smoking status, and study covariates were included (Fig. 1).

### 2.2. Measurement of smoking status, SHS exposure, CKD, and all-cause mortality

Never-smokers were defined as participants who reported smoking fewer than 100 cigarettes in their lifetime and no use of nicotine-containing products during the preceding 5 days. Because most active smokers have serum cotinine concentrations exceeding 10 ng/mL,^[12,13]^ SHS exposure was categorized as unexposed (<0.05 ng/mL), low exposure (0.05–1 ng/mL), moderate exposure (1–10 ng/mL), or heavy exposure (>10 ng/mL). Serum cotinine was measured using isotope-dilution high-performance liquid chromatography or atmospheric-pressure chemical ionization tandem mass spectrometry.^[14]^

Estimated glomerular filtration rate was calculated from serum creatinine using the Chronic Kidney Disease Epidemiology Collaboration 2021 equation.^[15]^ Urinary albumin-to-creatinine ratio was calculated as urinary albumin (mg/dL) divided by urine creatinine (g/dL). CKD was defined as estimated glomerular filtration rate <60 mL/min/1.73 m^2^ or urinary albumin-to-creatinine ratio >30 mg/g, consistent with the study definition based on Kidney Disease: Improving Global Outcomes guidance.^[16]^

Among participants with CKD, the prospective outcome was all-cause mortality, defined as death from any cause during follow-up. Vital status was ascertained from the NHANES-linked mortality files, and follow-up time was calculated from the examination date to death or the administrative censoring date.

### 2.3. Covariate definitions

Covariates included sex, age, race, educational level, marital status, alanine aminotransferase, aspartate aminotransferase, blood urea nitrogen, total cholesterol, body mass index (BMI), ratio of family income to poverty, alcohol consumption, diabetes, CVD, and hypertension.

Alcohol consumption was classified as none or moderate (0–1 drink/day for women or 0–2 drinks/day for men), heavy (2–3 drinks/day for women or 3–4 drinks/day for men), or binge (≥4 drinks/day for women or ≥5 drinks/day for men). Diabetes was defined by a physician diagnosis, fasting blood glucose ≥126 mg/dL, or glycated hemoglobin ≥6.5%. Hypertension was defined by a prior diagnosis, use of antihypertensive medication, or systolic/diastolic blood pressure ≥140/90 mm Hg. CVD was defined by a prior diagnosis of congestive heart failure, coronary artery disease, angina pectoris, or myocardial infarction. Additional covariates used in sensitivity analyses included partner/household smoking (SMQFAM), occupational exposure (OCQ290G; available for 1999–2010), a composite index incorporating volatile organic compounds and metals, Healthy Eating Index 2015, physical activity, CKD-related medications, and kidney disease history.

### 2.4. Statistical analyses

Continuous variables are summarized as mean ± standard deviation, and categorical variables as numbers and proportions. Associations between SHS exposure and CKD were examined using multivariable logistic regression. Serum cotinine was modeled both continuously after log_2_ transformation and categorically using the 4 exposure groups defined above. Smoothed curves were estimated using penalized splines within generalized additive models (GAMs) to assess potential nonlinearity. For all-cause mortality among participants with CKD, Cox proportional hazards models were used to estimate hazard ratios (HRs) and 95% confidence intervals (CIs). Subgroup analyses and interaction tests were used to evaluate the consistency of mortality associations across prespecified covariates. Sensitivity analyses included application of NHANES sampling weights in logistic and Cox regression models to account for the complex survey design; additional adjustment for partner/household smoking, occupational exposure, volatile organic compounds/metals, diet, physical activity, CKD-related medications, and kidney disease history; and competing-risk analyses for cause-specific mortality using Fine and Gray models. Because age appeared to be an important confounder in initial models, a post hoc analysis stratified participants at the median age of 44 years. Two-sided *P* values <.05 were considered statistically significant. Analyses were conducted using EmpowerStats (X&Y Solutions, Inc.) and R (R Foundation for Statistical Computing).

## 3. Results

### 3.1. Clinical characteristics of participants with and without CKD

Table 1 presents baseline characteristics of the 13,276 never-smoking adults, including 1630 participants (12.30%) who met the study definition of CKD. Participants with CKD were older and had a higher mean BMI than those without CKD (both *P* < .01). Mean serum cotinine was 10.80 ± 69.38 ng/mL in participants with CKD and 10.72 ± 66.70 ng/mL in those without CKD (*P* = .01). Participants with CKD also had higher blood urea nitrogen, lower alanine aminotransferase, and higher prevalences of hypertension, diabetes, and CVD. Race, educational level, marital status, and alcohol-consumption categories also differed by CKD status.

**Table 1:** Baseline characteristics according to the presence or absence of CKD in NHANES 1999 to 2020.

Variables	Total (n = 13,276)	No (n = 11,646)	Yes (n = 1630)	*P* value
Age, yr	44.41 ± 16.95	42.47 ± 15.74	58.26 ± 18.71	<.01
PIR	2.99 ± 1.64	3.02 ± 1.65	2.83 ± 1.58	<.01
Cotinine, ng/mL	10.73 ± 67.03	10.72 ± 66.70	10.80 ± 69.38	.01
ALT, U/L	25.13 ± 17.74	25.22 ± 17.94	24.46 ± 16.25	.02
AST, U/L	25.01 ± 16.77	24.91 ± 17.21	25.69 ± 13.22	<.01
BUN, mg/dL	13.20 ± 5.07	12.66 ± 4.09	17.10 ± 8.52	<.01
TC, mg/dL	195.04 ± 40.51	194.68 ± 39.85	197.60 ± 44.89	.07
BMI, kg/m^2^	28.86 ± 6.83	28.68 ± 6.69	30.14 ± 7.60	<.01
Sex (%)				.27
Male	5099 (45.94)	5371 (46.12)	728 (44.66)	
Female	7177 (54.06)	6275 (53.88)	902 (55.34)	
Race (%)				<.01
Mexican American	2407 (18.13)	2174 (18.67)	233 (14.29)	
Other races	2414 (18.18)	2167 (18.61)	247 (15.15)	
Non-Hispanic White	5851 (44.07)	5033 (43.22)	818 (50.18)	
Non-Hispanic Black	2604 (19.61)	2272 (19.51)	332 (20.37)	
Education (%)				<.01
<High school	2142 (16.13)	1792 (15.39)	350 (21.47)	
High school	2529 (19.05)	2155 (18.50)	374 (22.94)	
>High school	8605 (64.82)	7699 (66.11)	906 (55.58)	
Marital status (%)				<.01
Married or living with a partner	8304 (62.55)	7356 (63.16)	948 (58.16)	
Living alone	4972 (37.45)	4290 (36.84)	682 (41.84)	
Drinking (%)				<.01
None or moderate	9535 (71.82)	8261 (70.93)	1274 (78.16)	
Heavy	2398 (18.06)	2188 (18.79)	210 (12.88)	
Binge	1343 (10.12)	1197 (10.28)	146 (8.96)	
Hypertension (%)				<.01
No	9709 (73.13)	8953 (76.88)	756 (46.38)	
Yes	3567 (26.87)	2693 (23.12)	874 (53.62)	
Diabetes (%)				<.01
No	12,255 (92.31)	10,982 (94.30)	1273 (78.10)	
Yes	1021 (7.698)	664 (5.70)	357 (21.90)	
CVD (%)				<.01
No	12,693 (95.61)	11,320 (97.20)	1373 (84.23)	
Yes	583 (4.39)	326 (2.80)	257 (15.77)	
SHS exposure (%)				.21
Unexposed	8422 (63.44)	7350 (63.11)	1072 (65.77)	
Low exposure	3425 (25.80)	3028 (26.00)	397 (24.36)	
Moderate exposure	658 (4.96)	582 (5.00)	76 (4.66)	
Heavy exposure	771 (5.81)	686 (5.89)	85 (5.21)	

Data were mean ± SD or median (IQR) for skewed variables or numbers (proportions) for categorical variables.

ALT = alanine aminotransferase, AST = aspartate aminotransferase, BMI = body mass index, BUN = blood urea nitrogen, CKD = chronic kidney disease, CVD = cardiovascular disease, IQR = interquartile range, NHANES = National Health and Nutrition Examination Survey, PIR = ratio of family income to poverty, SD = standard deviation, SHS = secondhand smoke, TC = total cholesterol.

### 3.2. Relationships between SHS exposure and CKD

Table 2 summarizes logistic regression analyses of SHS exposure and CKD prevalence. In the fully adjusted model, moderate SHS exposure was associated with higher CKD prevalence (odds ratio [OR] = 1.37, 95% CI: 1.07–1.76; *P* = .01) compared with the unexposed group. Low exposure (OR = 1.01, 95% CI: 0.89–1.15; *P* = .90) and heavy exposure (OR = 1.14, 95% CI: 0.89–1.45; *P* = .30) were not significantly associated with CKD. Log_2_-transformed cotinine was also not significantly associated with CKD after full covariate adjustment (OR = 1.01, 95% CI: 0.99–1.03; *P* = .21).

**Table 2:** The relationships between SHS exposure and CKD among never-smoking adults.

Variables	Model 1	Model 2	Model 3
	OR (95% CI), *P* value	OR (95% CI), *P* value	OR (95% CI), *P* value
Log_2_-transformed cotinine, ng/mL	0.98 (0.96–0.99), <.01	1.03 (1.01–1.05), <.01	1.01 (0.99–1.03), .21
SHS exposure			
Unexposed	Reference	Reference	Reference
Low exposure	0.87 (0.78–0.98), .02	1.14 (1.01–1.28), .04	1.01 (0.89–1.15), .90
Moderate exposure	0.95 (0.76–1.19), .67	1.68 (1.33–2.14), <.01	1.37 (1.07–1.76), .01
Heavy exposure	0.78 (0.63–0.97), .03	1.31 (1.04–1.65), .02	1.14 (0.89–1.45), .30

Model 1: unadjusted.

Model 2: adjusted for sex, age, and race.

Model 3: adjusted for sex, age, race, education, body mass index, ratio of family income to poverty, marital status, hypertension, drinking, diabetes, and cardiovascular disease.

CI = confidence interval, CKD = chronic kidney disease, OR = odds ratio, SHS = secondhand smoke.

**Figure 2.**
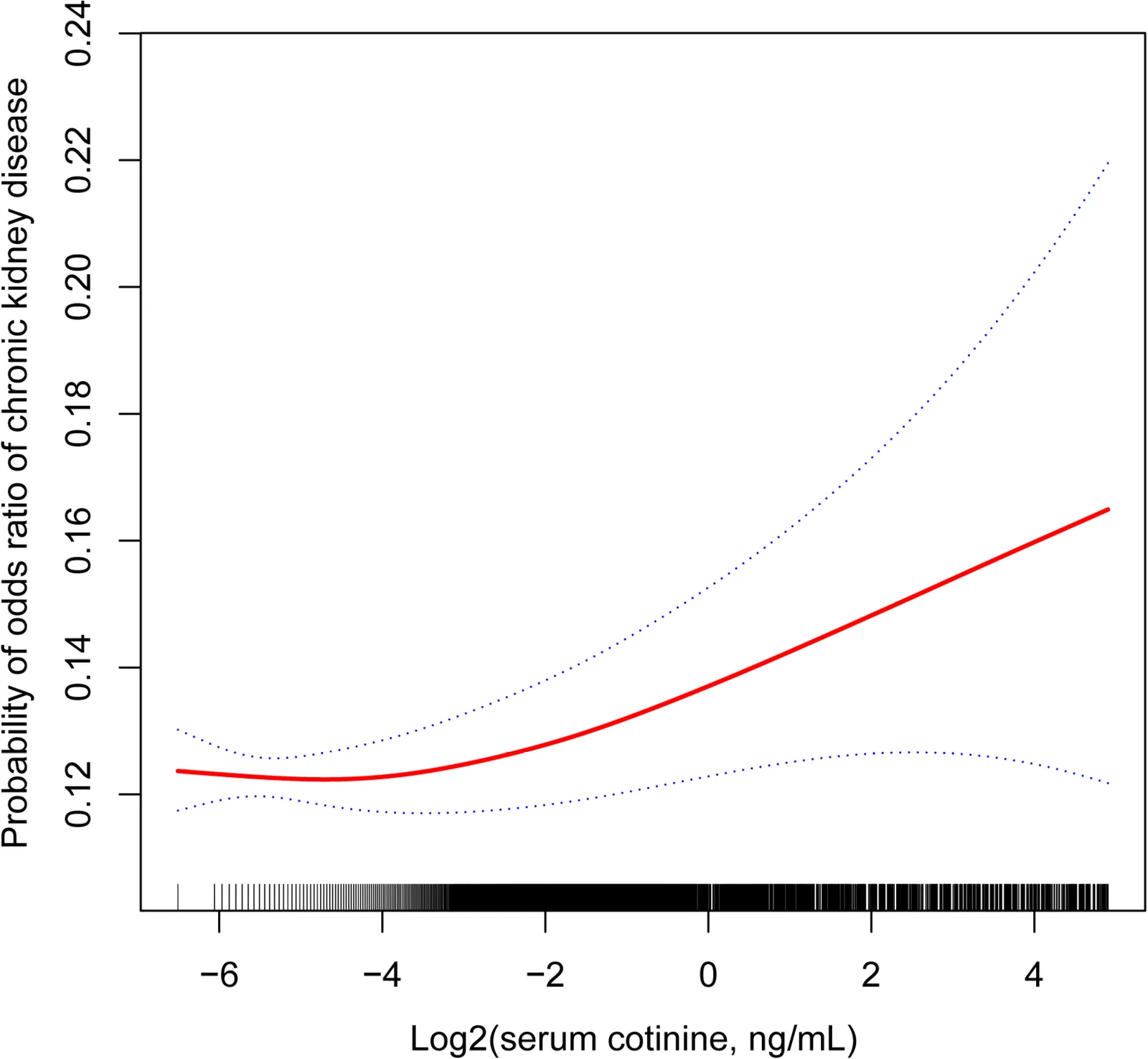
Dose-response relationship between SHS exposure and CKD among never-smoking adults. The model was adjusted for sex, age, race, education, body mass index, ratio of family income to poverty, marital status, hypertension, drinking, diabetes, and cardiovascular disease. The red and blue areas represent estimated values and corresponding 95% confidence intervals, respectively; 100% of the data are shown. CKD = chronic kidney disease, SHS = secondhand smoke.

Figure 2 shows the GAM curve for the association between log_2_-transformed serum cotinine and CKD prevalence.

In post hoc age-stratified analyses, moderate SHS exposure was associated with CKD among adults aged ≥44 years (fully adjusted OR = 1.84, 95% CI: 1.33–2.55; *P* < .01), whereas no statistically significant association was observed in the younger stratum. These analyses are presented in Table S4, Supplemental Digital Content 1 and Figure S2, Supplemental Digital Content 2.

### 3.3. Relationships between SHS exposure and all-cause mortality

The median follow-up duration for the mortality cohort was 7.8 years. In fully adjusted analyses of never-smoking adults with CKD (Table 3), each doubling of serum cotinine concentration was associated with a 4% higher hazard of all-cause mortality (HR = 1.04, 95% CI: 1.01–1.08; *P* = .02). Compared with the unexposed group, moderate SHS exposure was associated with higher all-cause mortality (HR = 2.03, 95% CI: 1.23–3.36; *P* < .01), as was heavy exposure (HR = 1.80, 95% CI: 1.11–2.93; *P* = .02). Low exposure was not significantly associated with mortality (HR = 0.98, 95% CI: 0.76–1.27; *P* = .86).

**Table 3:** The relationships between SHS exposure and all-cause mortality among never-smoking adults with CKD.

Variables	Model 1	Model 2	Model 3
	HR (95% CI), *P* value	HR (95% CI), *P* value	HR (95% CI), *P* value
Log_2_-transformed cotinine, ng/mL	1.00 (0.97–1.04), .99	1.06 (1.03–1.10), <.01	1.04 (1.01–1.08), .02
SHS exposure			
Unexposed	Reference	Reference	Reference
Low exposure	0.73 (0.57–0.93), .01	1.00 (0.78–1.28), .98	0.98 (0.76–1.27), .86
Moderate exposure	1.10 (0.70–1.74), .69	2.41 (1.50–3.86), <.01	2.03 (1.23–3.36), <.01
Heavy exposure	1.28 (0.82–1.99), .29	2.08 (1.31–3.30), <.01	1.80 (1.11–2.93), .02

Model 1: unadjusted.

Model 2: adjusted for sex, age, and race.

Model 3: adjusted for sex, age, race, education, body mass index, ratio of family income to poverty, marital status, hypertension, ALT, AST, BUN, TC, drinking, diabetes, and cardiovascular disease.

ALT = alanine aminotransferase, AST = aspartate aminotransferase, BUN = blood urea nitrogen, CI = confidence interval, CKD = chronic kidney disease, HR = hazard ratio, SHS = secondhand smoke, TC = total cholesterol.

**Figure 3.**
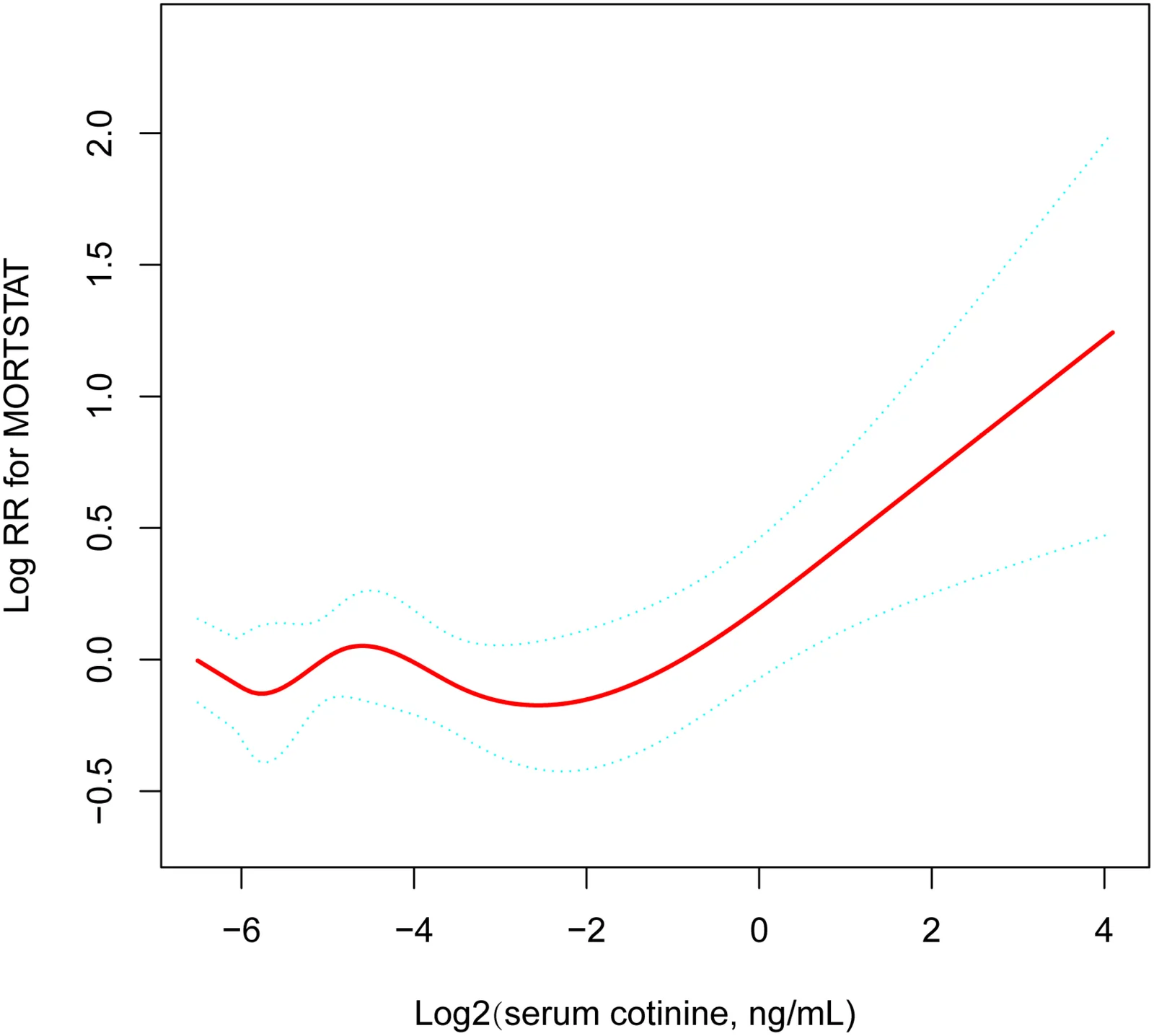
Dose-response relationship between SHS exposure and all-cause mortality among never-smoking adults with CKD. The model was adjusted for sex, age, race, education, body mass index, ratio of family income to poverty, marital status, hypertension, ALT, AST, BUN, TC, drinking, diabetes, and cardiovascular disease. The red and blue areas represent estimated values and corresponding 95% confidence intervals, respectively; 100% of the data are shown. ALT = alanine aminotransferase, AST = aspartate aminotransferase, BUN = blood urea nitrogen, CKD = chronic kidney disease, MORTSTAT = mortality status, RR = risk ratio, SHS = secondhand smoke, TC = total cholesterol.

As shown in Figure 3, the GAM analysis did not suggest clear nonlinearity and was compatible with an overall positive association between serum cotinine and all-cause mortality.

### 3.4. Subgroup analyses of SHS exposure and all-cause mortality

Subgroup analyses are shown in Figure S1, Supplemental Digital Content 3. There was no evidence of interaction by sex (*P* = .79), age (*P* = .66), race (*P* = .15), education (*P* = .72), ratio of family income to poverty (*P* = .69), drinking status (*P* = .30), BMI (*P* = .73), diabetes (*P* = .58), CVD (*P* = .85), or hypertension (*P* = .73). An interaction was observed for marital status (*P* = .01), with a positive association among married/partnered participants but not among those living alone. This isolated interaction should be interpreted cautiously in the context of multiple subgroup comparisons.

### 3.5. Sensitivity analyses

Sensitivity analyses are presented in Table S1, Supplemental Digital Content 4. Weighted and expanded-covariate logistic models for CKD prevalence are shown in Table S1, Supplemental Digital Content 4, and corresponding Cox models for all-cause mortality are shown in Table S2, Supplemental Digital Content 5. These analyses were generally directionally compatible with the primary findings, although estimates were less precise in the more extensively adjusted models. Cause-specific competing-risk analyses are shown in Table S3, Supplemental Digital Content 6; associations with cardiovascular and cancer mortality were not statistically significant, while a positive association with other-cause mortality was observed for log_2_-transformed cotinine and moderate SHS exposure.

## 4. Discussion

In this analysis of 13,276 never-smoking adults, moderate SHS exposure was associated with higher CKD prevalence after adjustment for demographic, socioeconomic, and clinical covariates. Among 1630 participants with CKD, moderate and heavy SHS exposure were associated with higher all-cause mortality, and each doubling of serum cotinine was associated with a 4% higher mortality hazard. The CKD analysis was cross-sectional, whereas the mortality analysis was prospective; accordingly, the findings should be interpreted as associations rather than evidence of causality.

Our findings extend previous work linking SHS exposure with CKD.^[2,7]^ The present study used serum cotinine to characterize exposure and additionally examined all-cause mortality among participants with established CKD. Over a median follow-up of 7.8 years, moderate and heavy exposure categories were associated with higher mortality. This extension from CKD prevalence to prognosis provides complementary evidence that biomarker-defined SHS exposure may identify a clinically vulnerable subgroup, while the observational design precludes conclusions about a direct protective or harmful causal effect of any specific exposure category.

Several biological pathways could plausibly contribute to the observed associations. Prior studies have linked tobacco-smoke exposure with oxidative stress, vascular injury, inflammatory responses, and renal dysfunction.^[17–19]^ Cotinine-based exposure assessment has also been used in studies examining systemic and kidney-related consequences of SHS.^[9,20,21]^ Other observational studies have reported associations between cotinine measures, blood pressure, and CKD-related outcomes across different populations.^[5,22,23]^ SHS may promote systemic inflammation and immune dysregulation,^[24,25]^ processes that could be especially relevant in CKD, where cardiovascular and infectious complications contribute substantially to adverse outcomes.^[2,26,27]^ These mechanisms remain hypotheses in the context of the present observational analysis and were not directly tested.

Age was an important confounder in the association between SHS exposure and CKD. In post hoc stratified analyses, the association with moderate exposure was observed among participants aged ≥44 years but not among younger adults. However, the age interaction test did not establish statistically significant effect modification; therefore, the age-stratified results should be viewed as exploratory rather than as evidence of a definitive age-specific effect.

This study has several strengths, including a large population-based sample, objective biomarker-based assessment of recent tobacco-smoke exposure, evaluation of both CKD prevalence and subsequent mortality, and multiple sensitivity analyses. Several limitations should also be considered. First, the CKD analysis was cross-sectional and cannot establish temporal or causal relationships. Second, residual confounding remains possible despite multivariable and expanded-covariate adjustment. Third, serum cotinine reflects relatively recent exposure and may not capture intermittent or long-term SHS exposure, creating potential exposure misclassification. Fourth, information on factors such as smoke-free policies, ventilation, and ambient PM2.5 was not available. Fifth, the mortality analysis was restricted to participants with CKD and had more limited precision in extensively adjusted sensitivity models. Finally, the findings may not generalize to individuals younger than 20 years or to populations outside the NHANES sampling frame.

## 5. Conclusion

Among never-smoking adults in NHANES, moderate SHS exposure was associated with higher CKD prevalence, and among participants with CKD, higher SHS exposure was associated with greater all-cause mortality. These findings support continued efforts to reduce SHS exposure and warrant confirmation in prospective studies designed to characterize long-term exposure and clarify temporality and mechanisms.

## Acknowledgments

The authors thank the participants and staff of NHANES for their contributions to data collection.

## Author contributions

**Conceptualization:** Renjie Huang, Miao Huang, Rui Zhang.

**Data curation:** Renjie Huang, Miao Huang.

**Formal analysis:** Renjie Huang.

**Funding acquisition:** Rui Zhang.

**Writing – original draft:** Renjie Huang, Miao Huang.

**Writing – review & editing:** Rui Zhang.












