# Supplementary material for: Secondhand smoke exposure in never-smokers: A cross-sectional and prospective cohort study of chronic kidney disease prevalence and all-cause mortality in affected patients
Source: Medicine (Baltimore). 2026 Sep 18;105(38):e50614. doi: 10.1097/MD.0000000000050614 (PMC13593294; doi:10.1097/MD.0000000000050614)
Supplement: Supplementary file 1 [file medi-105-e50614-s002.pdf]

Supplemental Digital Content 6

Fig. S2 Subgroup analysis examining the relationship between SHS exposure and CKD among never-smoking adults.

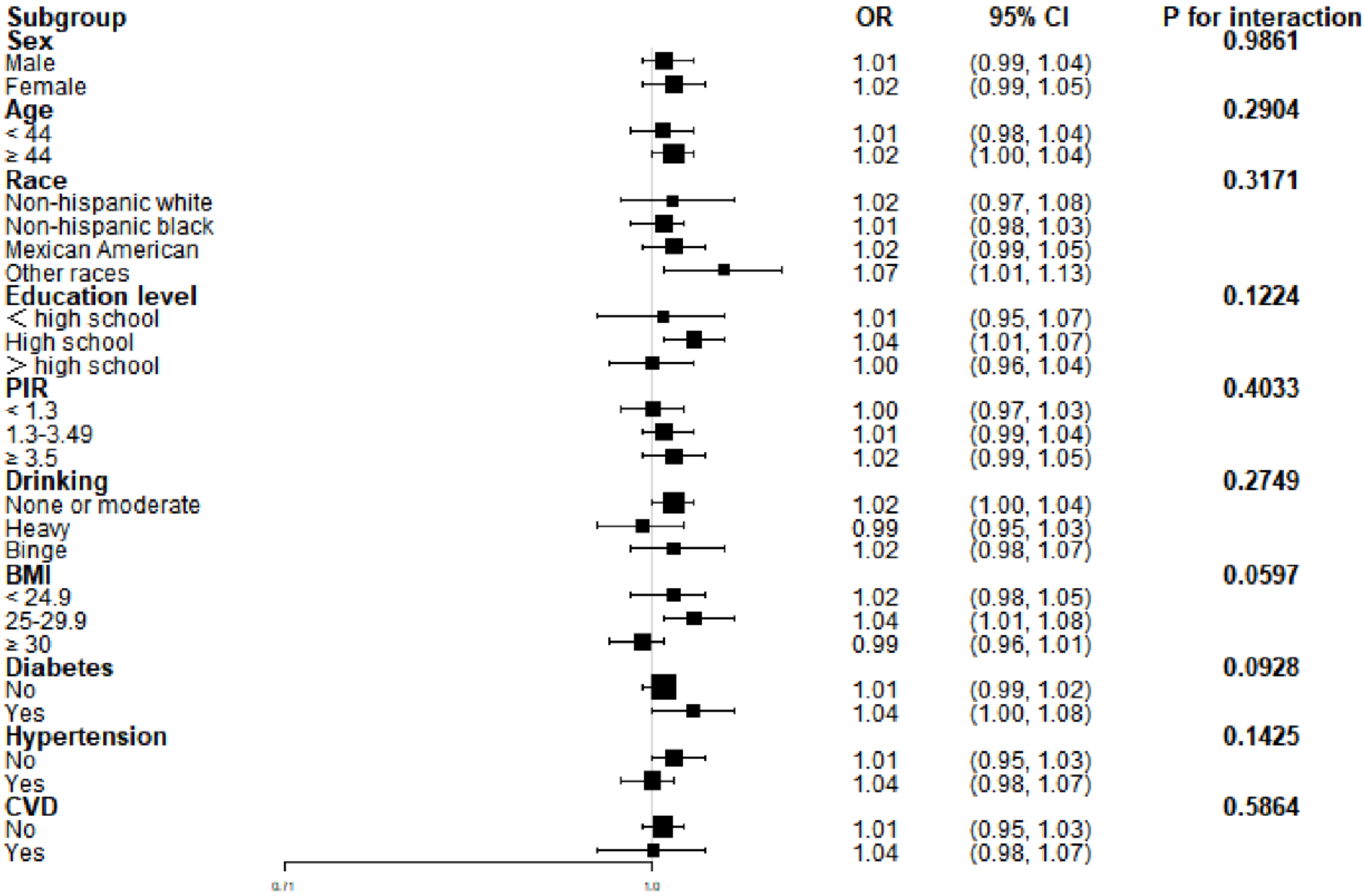

Statistical tests: Model 3 adjusted for all covariates. Model 3: adjusted by sex, age, race, education, body mass index, ratio of family income to poverty, marital status, hypertension, drinking, diabetes and cardiovascular disease.

OR odds ratio, CI confidence interval, SHS secondhand smoke, CKD chronic kidney disease, PIR Ratio of family income to poverty, BMI body mass index, CVD cardiovascular disease.
