# Supplementary material for: Secondhand smoke exposure in never-smokers: A cross-sectional and prospective cohort study of chronic kidney disease prevalence and all-cause mortality in affected patients
Source: Medicine (Baltimore). 2026 Sep 18;105(38):e50614. doi: 10.1097/MD.0000000000050614 (PMC13593294; doi:10.1097/MD.0000000000050614)
Supplement: Supplementary file 3 [file medi-105-e50614-s001.docx]

**Supplemental Digital Content 4**

**Table S4 The relationships between SHS exposure and CKD among never-smoking adults stratified by age.**

| Variables | Model1  OR (95% CI) *P* value | Model2  OR (95% CI) *P* value | Model3  OR (95% CI) *P* value |
| --- | --- | --- | --- |
| Age <44 | | | |
| Log2-transformed cotinine, ng/mL | 1.00 (0.97, 1.02) 0.81 | 1.02 (0.99, 1.04) 0.28 | 1.01 (0.98, 1.04) 0.55 |
| *SHS exposure* |  |  |  |
| Unexposed | Reference | Reference | Reference |
| Low exposure | 0.97 (0.78, 1.22) 0.81 | 1.07 (0.85, 1.34) 0.59 | 0.97 (0.76, 1.23) 0.79 |
| Moderate exposure | 0.85 (0.56, 1.29) 0.44 | 0.98 (0.64, 1.49) 0.91 | 0.91 (0.58, 1.41) 0.66 |
| Heavy exposure | 1.15 (0.82, 1.61) 0.43 | 1.42 (0.99, 2.03) 0.06 | 1.35 (0.93, 1.97) 0.11 |
| Age ≥44 | | | |
| Log2-transformed cotinine, ng/mL | 1.02 (1.00, 1.03) 0.08 | 1.04 (1.02, 1.06) <0.01 | 1.02 (1.00, 1.04) 0.12 |
| *SHS exposure* |  |  |  |
| Unexposed | Reference | Reference | Reference |
| Low exposure | 1.02 (0.89, 1.17) 0.79 | 1.15 (0.99, 1.33) 0.07 | 1.03 (0.88, 1.20) 0.72 |
| Moderate exposure | 1.77 (1.33, 2.35) <0.01 | 2.28 (1.68, 3.10) <0.01 | 1.84 (1.33, 2.55) <0.01 |
| Heavy exposure | 1.01 (0.76, 1.35) 0.95 | 1.19 (0.87, 1.63) 0.27 | 1.03 (0.74, 1.43) 0.84 |

Model 1: unadjusted.

Model 2: adjusted by sex and race.

Model 3: adjusted by sex, race, education, body mass index, ratio of family income to poverty, marital status, hypertension, drinking, diabetes and cardiovascular disease

OR odds ratio, CI confidence interval, SHS secondhand smoke, CKD chronic kidney disease.
