# Supplementary material for: Secondhand smoke exposure in never-smokers: A cross-sectional and prospective cohort study of chronic kidney disease prevalence and all-cause mortality in affected patients
Source: Medicine (Baltimore). 2026 Sep 18;105(38):e50614. doi: 10.1097/MD.0000000000050614 (PMC13593294; doi:10.1097/MD.0000000000050614)
Supplement: Supplementary file 4 [file medi-105-e50614-s004.docx]

**Supplemental Digital Content 1**

**Table S1 The relationships between SHS exposure and CKD among never-smoking adults.**

| Variables | Model1  OR (95% CI) *P* value | Model2  OR (95% CI) *P* value | Model3  OR (95% CI) *P* value |
| --- | --- | --- | --- |
| Log2-transformed cotinine, ng/mL | 0.97 (0.97, 0.98) <0.01 | 0.98 (0.98, 0.99) <0.01 | 1.01 (0.99, 1.04) 0.41 |
| *SHS exposure* |  |  |  |
| Unexposed | Reference | Reference | Reference |
| Low exposure | 0.95 (0.90, 0.99) 0.02 | 1.10 (1.05, 1.16) <0.01 | 1.00 (0.82, 1.22) 0.99 |
| Moderate exposure | 0.86 (0.79, 0.93) <0.01 | 1.16 (1.06, 1.27) <0.01 | 1.22 (0.83, 1.78) 0.31 |
| Heavy exposure | 0.69 (0.66, 0.73) <0.01 | 0.75 (0.71, 0.79) <0.01 | 1.45 (0.71, 2.95) 0.31 |

Model 1: unadjusted.

Model 2: adjusted by sex, age and race.

Model 3: adjusted by sex, age, race, education, body mass index, ratio of family income to poverty, marital status, hypertension, drinking, diabetes, cardiovascular disease, partner/household smoking, occupational exposure, volatile organic compounds, metals, diet, physical activity, CKD-related medications, and kidney disease

OR odds ratio, CI confidence interval, SHS secondhand smoke, CKD chronic kidney disease.
