# Supplementary material for: Secondhand smoke exposure in never-smokers: A cross-sectional and prospective cohort study of chronic kidney disease prevalence and all-cause mortality in affected patients
Source: Medicine (Baltimore). 2026 Sep 18;105(38):e50614. doi: 10.1097/MD.0000000000050614 (PMC13593294; doi:10.1097/MD.0000000000050614)
Supplement: Supplementary file 5 [file medi-105-e50614-s005.docx]

**Supplemental Digital Content 2**

**Table S2 The relationships between SHS exposure and all-cause mortality among never-smoking adults with CKD.**

| Variables | Model1  HR (95% CI) *P* value | Model2  HR (95% CI) *P* value | Model3  HR (95% CI) *P* value |
| --- | --- | --- | --- |
| Log2-transformed cotinine, ng/mL | 0.98 (0.95, 1.01) 0.20 | 1.03 (1.01, 1.06) 0.02 | 1.19 (1.03, 1.39) 0.02 |
| *SHS exposure* |  |  |  |
| Unexposed | Reference | Reference | Reference |
| Low exposure | 0.79 (0.64, 0.99) 0.04 | 1.13 (0.91, 1.41) 0.27 | 2.29 (0.73, 7.20) 0.16 |
| Moderate exposure | 0.97 (0.63, 1.50) 0.89 | 1.46 (0.94, 2.27) 0.10 | 15.57 (1.50, 161.73) 0.02 |
| Heavy exposure | 0.82 (0.55, 1.22) 0.33 | 1.49 (1.00, 2.24) 0.05 | 7.62 (1.13, 51.42) 0.04 |

Model 1: unadjusted.

Model 2: adjusted by sex, age and race.

Model 3: adjusted by sex, age, race, education, body mass index, ratio of family income to poverty, marital status, hypertension, ALT, AST, BUN, TC, drinking, diabetes, cardiovascular disease, partner/household smoking, occupational exposure, volatile organic compounds, metals, diet, physical activity, CKD-related medications, and kidney disease.

HR hazard ratio, CI confidence interval, SHS secondhand smoke, CKD chronic kidney disease, ALT alanine aminotransferase, AST aspartate aminotransferase, BUN blood urea nitrogen, TC total cholesterol.
